# Role of en-APTAS Membranes in Enhancing the NO_2_ Gas-Sensing Characteristics of Carbon Nanotube/ZnO-Based Memristor Gas Sensors

**DOI:** 10.3390/bios14120635

**Published:** 2024-12-20

**Authors:** Ibtisam Ahmad, Mohsin Ali, Hee-Dong Kim

**Affiliations:** Department of Semiconductor Systems Engineering, Convergence Engineering for Intelligent Drone, Institute of Semiconductor and System IC, Sejong University, 209, Neungdong-ro, Gwangjin-gu, Seoul 05006, Republic of Korea

**Keywords:** carbon nanotubes, N-[3-(trimethoxysilyl)propyl] ethylene diamine, gas sensor, conduction filament heater

## Abstract

NO_2_ is a toxic gas that can damage the lungs with prolonged exposure and contribute to health conditions, such as asthma in children. Detecting NO_2_ is therefore crucial for maintaining a healthy environment. Carbon nanotubes (CNTs) are promising materials for NO_2_ gas sensors due to their excellent electronic properties and high adsorption energy for NO_2_ molecules. However, conventional CNT-based sensors face challenges, including low responses at room temperature (RT) and slow recovery times. This study introduces a memristor-based NO_2_ gas sensor comprising CNT/ZnO/ITO decorated with an N-[3-(trimethoxysilyl)propyl] ethylene diamine (en-APTAS) membrane to enhance room-temperature-sensing performance. The amine groups in the en-APTAS membrane increase adsorption sites and boost charge transfer interactions between NO_2_ and the CNT surface. This modification improves the sensor’s response by 60% at 20 ppm compared to the undecorated counterpart. However, the high adsorption energy of NO_2_ slows the recovery process. To overcome this, a pulse-recovery method was implemented, applying a −2.5 V pulse with a 1 ms width, enabling the sensor to return to its baseline within 1 ms. These findings highlight the effectiveness of en-APTAS decoration and pulse-recovery techniques in improving the sensitivity, response, and recovery of CNT-based gas sensors.

## 1. Introduction

The ability to detect specific gas types is crucial across various sectors, including food safety, healthcare, pollution monitoring, and industrial process management. Thus, researchers are working on highly sensitive and selective gas sensors to detect these gases. NO_2_ is a toxic pollutant that is produced as a byproduct in industry and emitted from vehicle exhausts, causing serious health issues such as asthma and bronchitis [1]. Therefore, the detection of NO_2_ gas is required so that its concentration can be evaluated in an effort to maintain a healthy habitat. Recently, there have been various studies and efforts to fabricate a NO_2_ gas sensor based on metal oxides [2], conducting polymers [3], and carbon-based materials [4]. Among these carbon materials, carbon nanotubes (CNTs) have been extensively studied for their superlative properties such as high specific surface area, high carrier mobility, and high detection sensitivity to NO_2_ gas at room temperature (RT) [5]. Despite this, the CNTs-based gas sensor still faces issues related to stability due to surface poisoning or high gas diffusion of NO_2_ gas into CNTs. This high diffusion energy results in the irreversible change in the resistance state and a very slow recovery process [6]. To address these challenges, various methods and techniques have been employed, utilizing external energy sources to modify the operating conditions of CNT-based gas sensors. These techniques include the use of ultraviolet light [7], visible light [8], and external heaters [9]. However, the use of an external light source, such as UV light or a visible-light-emitting diodes (LEDs), requires integrating additional components into the sensor system. This integration increases the device’s overall energy consumption and adds complexity to its design and operation [10]. Furthermore, external heating systems are required to attain the high operational temperature for traditional gas sensors to attain maximum efficiency. Thus, the elevated operational temperature leads to high power consumption [11]. Therefore, it is important to further look for possible solutions to develop systems that can improve gas sensor technology.

Memristors offer a range of advantages regarding gas-sensing applications, combining sensitivity, efficiency, and versatility. Memristors retain their state without constant energy input, unlike conventional sensors, which often require a continuous supply of power in order to maintain their operation [12,13]. Memristor-based gas sensors exhibit exceptional sensitivity by detecting traces of gas concentrations with precision, and their nanoscale dimensions enable miniaturization and integration into advanced devices. Memristor-based gas sensors also provide rapid response and recovery, which ensure real-time monitoring in dynamic environments [13]. Their compatibility with diverse materials allows for the tailored sensing of specific gases, whereas their robustness ensures reliable performance under extreme conditions. Memristors also integrate sensing, data processing, and storage, which eliminate the need for external circuitry and support intelligent functionalities, such as pattern recognition [14]. Cost-effective fabrication further enhances their viability, making memristors a superior alternative to conventional gas sensors for modern applications.

On the other hand, the high sensitivity of gas sensors is crucial to the fulfillment of the requirements of various systems. Various approaches have been utilized to enhance the sensitivity of gas sensors, such as the development of heterostructure-based gas sensors [15], surface functionalization [16], composite material [17], and doping [18]. Therefore, the functionalization of the gas sensor surface with an en-APTAS membrane is reported to be an effective approach to improving sensitivity towards NOx gases. Chae et al. has reported the improved gas-sensing response of a CNTs gasistor towards NO gas by functionalizing the CNTs surface with en-APTAS [19]. Furthermore, Namsoo et al. reported an en-APTAS-decorated CNT gas sensor with improved gas-sensing response characteristics towards NO gas. However, the study did not cover the selectivity properties of the proposed gas sensor [20].

**Figure 1:**
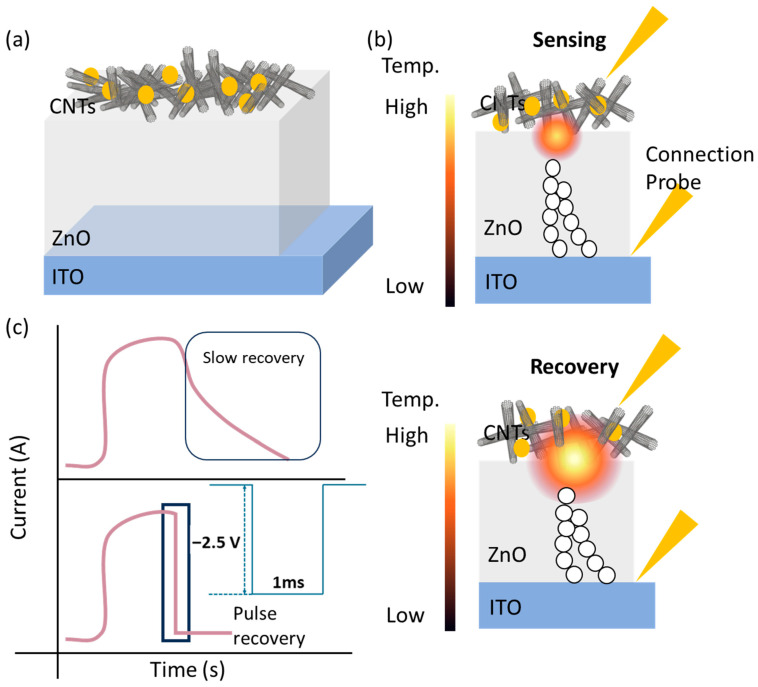
Schematic illustration of (**a**) en-APTAS-decorated CNTs-CFH gas sensor; (**b**) sensing mode in HRS; (**c**) pulse-recovery process. The pink line shows the schematics of transient response curve; the blue line shows a pulse with an amplitude of −2.5 V with a pulse width of 1 ms and recovery mode in HRS.

We propose an en-APTAS-decorated CNTs/ZnO/ITO-based memristor gas sensor in this study for the detection of NO₂ gas, as shown in Figure 1a. Here, en-APTAS is used for the functionalization of the CNTs surface in order to obtain enhanced sensitivity of a CNTs/ZnO/ITO memristor-based gas sensor. It is consequently observed that the en-APTAS-decorated CNTs/ZnO/ITO gas sensor showed an enhanced gas-sensing response to NO_2_ gas at 20–0.5 ppm compared to a bare CNTs/ZnO/ITO gas sensor. The sensing voltage (V_sensing_) is set to 0.5 V in order to observe the gas-sensing characteristics at RT, which was optimized in our previous study [21]. Furthermore, ZnO is introduced as a conduction filament heater (CFH), providing thermal energy to activate the CNTs-based gas sensor. The CFH acts as a nano-heater, which works in the high-resistance state (HRS). The HRS of the CFH is obtained by applying the reset operation, which is driven by the Joule heating effect. The CFH works as a nano-heater due to power dissipation across the gap of the ruptured CF when a V_sensing_ of 0.5 V is applied to the proposed gas sensor, as shown in Figure 1b. Moreover, a pulse-recovery process is employed using the CFH, wherein a high-voltage pulse of −2.5 V with a pulse width of 1 ms is applied to the proposed gas sensor. The high voltage pulse generated a sufficient energy to enable the rapid desorption of NO₂ gas from the CNTs’ surface, and the proposed gas sensor attains the initial current level, as displayed in Figure 1c.

## 2. Materials and Methods

### 2.1. Dispersion of CNTs

CNTs are used as sensing material and deposited on the top of CFH. Prior to the deposition of CNTs, a dispersion of CNTs was prepared by mixing 0.2 mg of CNTs into 10 mL of benzene (SAMCHUM 99.0%, SAMCHUN Pure Chemical Co., Ltd., Seoul, Republic of Korea). The dispersion was then treated in an ultrasonic bath for 5 h, maintaining a sonication power of 70%, to achieve a uniform dispersion.

### 2.2. Fabrication of CNTs-CFH Gas Sensor

Prior to the fabrication process, ITO-coated glass substrates were cleaned using the standard cleaning process. Then, 100 nm of ZnO layer was deposited using radio frequency (RF) sputtering (KVS-2000L, vacuum system, and components were obtained from KOREA VACUUM TECH., Paju, Republic of Korea). Before the deposition process, the initial pressure of 1 uTorr was maintained. During the deposition process, 20 sccm Ar gas was utilized as a carrier gas and a working pressure was maintained at 5 mTorr. The photolithography process was performed using a chrome mask with a diameter of 200 µm to obtain the patterning for the top electrode (TE). CNTs were deposited as the TE onto the ZnO layer using the optimal dipping process previously reported in the literature [22]. Lift-off was performed using acetone to remove the photoresist and obtain the patterned CNTs. The deposited CNTs appeared as accumulated black dots on the top of the ZnO layer.

### 2.3. Fabrication of en-APTAS-Decorated CNTs-CFH Gas Sensor

Prior to the decoration process, an en-APTAS membrane (Sigma Aldrich 97%, Dow Coming Corp., Washington, DC, USA) solution was prepared in ethanol (SAMCHUM 99.5%, SAMCHUN Pure Chemical Co., Ltd., Republic of Korea) by adding 1 wt% en-APTAS into 99 wt% ethanol followed by a 15 min ultrasonication process to obtain a homogeneous solution. To perform the decoration process, the CNTs-coated CFH gas sensor was immersed in the solution for 10 min. Afterwards, the en-APTAS-decorated CNTs-CFH gas sensor was dried with N_2_ gas to remove any remaining traces of ethanol.

### 2.4. Measurements of CNTs-CFH Gas Sensor

Firstly, electrical measurements of CNTs-CFH were performed to obtain the RS characteristics using a Keithley 2400 SCS (Tektronix, I.V Solutions, Seoul, Republic of Korea. Gas sensing measurements were performed to obtain the response characteristics towards NO_2_ gas. All the measurements were performed at room temperature and a relative humidity of 30%. The gas sensing measurements were performed by injecting a constant flow of 500 sccm gas into the testing chamber. The gas flow was maintained using a microflow meter (MFC: DFPC1000). Dry air was used as a mixture of gas to obtain a constant flow of gas during the gas sensing measurements.

The proposed gas sensors were exposed to base gas for 50 s to maintain the baseline current. The target gas was injected for 150 s to measure the gas sensing characteristics. To calculate the response percentage of the CNTs-CFH gas sensor, the following equation was used [23].

(1) $$\mathrm{Response}\;(\%)=\left(\frac{I_{g}-I_{a}}{I_{a}}\right)\times 100$$

Here, I_g_ is the current in the target gas environment and I_a_ is the current in the base gas environment.

### 2.5. COMSOL Multiphysics Simulation

To gain a deeper insight into the gas sensing performance of the CFH-based CNT gas sensor, the heat distribution within the proposed CFH-based CNT gas sensor was analyzed using COMSOL Multiphysics 6.2. A Joule heating module was employed in the thermal field simulation to model both the thermal and electrical behaviors of the device. In the simulation, a bias voltage of 0.5 V was applied to the top electrode (TE), while the bottom electrode was grounded to calculate the thermal distribution. For accurate simulation results, the following continuity equations were applied:

(2) ∇⋅J=Q(j,v)

(3) $$J=\left(\sigma +\epsilon_{0}\epsilon_{r}\frac{\partial }{\partial t}\right)E+J_{e}$$

(4) E=−∇V

Here, J represents the current density, Qj, v denotes the heat source, V is the applied voltage, E refers to the electric field, and σ signifies the electrical conductivity. The material parameters utilized for these simulations are provided in Table 1. This analysis highlights the thermal dynamics essential for understanding and optimizing the device’s performance.

## 3. Results and Discussions

### 3.1. CFH-CNT Gas Sensor Simulations via COMSOL Multiphysics

**Figure 2:**
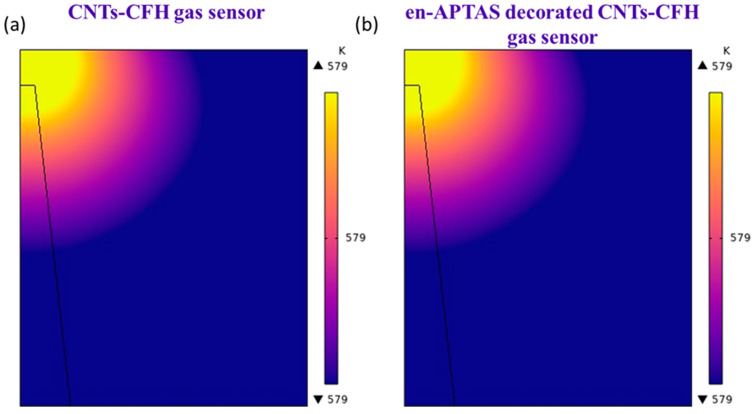
(**a**) COMSOL simulation of CNTs-CFH gas sensor; (**b**) en-APTAS-decorated CNTs-CFH gas sensor.

To evaluate the heating characteristics of the proposed CFH, heating profile was initially examined by using COMSOL Multiphysics 6.2 simulations. Heat generation in the CFH occurs in the HRS, where the maximum power dissipation occurs across the gap of ruptured CF due to the applied voltage. It is important to note that the CF is formed within the CFH through DC voltage sweeping in the positive bias region. Conversely, during the reverse operation, the CF ruptures due to Joule heating, particularly at the edge of the CF. This ruptured CF was utilized as the heat source in the COMSOL Multiphysics 6.2 simulations involving the CFH. The majority of the electric field is concentrated within the thin ZnO gap between the ruptured CF and the top electrode (TE), illustrating the electric field distribution when a V_sensing_ of 0.5 V is applied. This is one of the key outcomes of the simulation. Figure 2a,b present the simulated temperature distribution for the HRS of the CNTs-CFH gas sensor and the en-APTAS-coated CNTs-CFH gas sensor, respectively. In both cases, heat generated has the same value of 579 K. The reason to obtain the same temperature is due to the constant V_sensing_ of 0.5 V. Hence, the enhanced gas sensing response observed is mainly due to decoration of en-APTAS membrane on the surface of CNTs. In conclusion, the heating temperature generated in the CFH acts as a nano-heater for CNT gas sensors.

### 3.2. Material Characteristics

**Figure 3:**
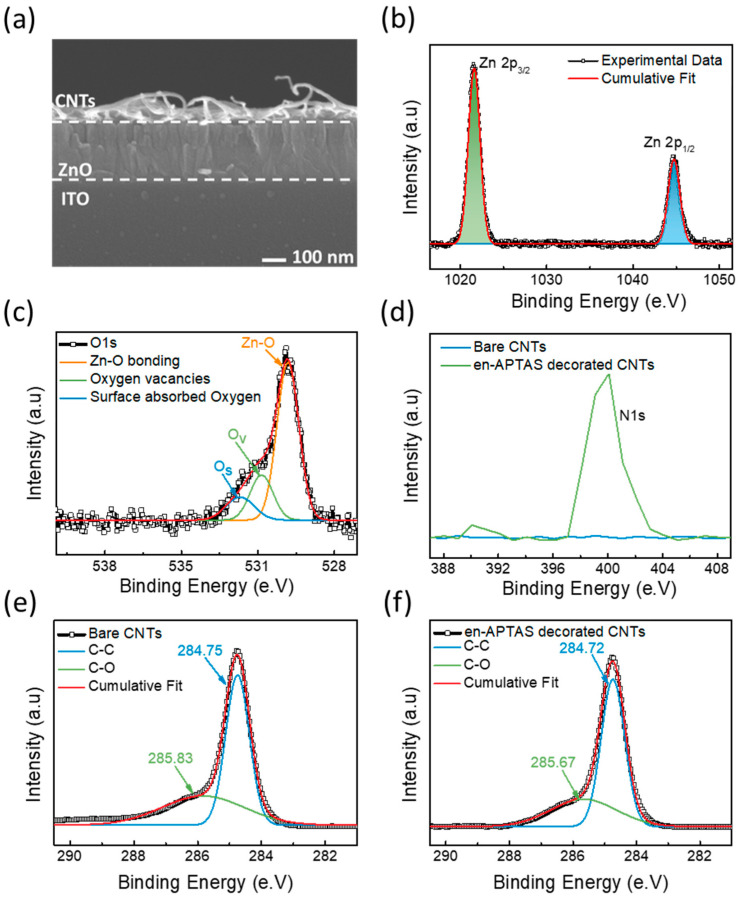
(**a**) Cross-sectional FE-SEM image of device. High-resolution XPS spectra of (**b**) Zn 2P and (**c**) O 1s from ZnO film. (**d**) XPS spectra of N1s peak with bare CNTs and en-APTAS-decorated CNTs; (**e**) XPS spectra of C 1s peaks of bare CNTs sample and (**f**) en-APTAS-decorated CNTs.

Material characterizations are performed to evaluate the structure and morphological properties of CNTs and ZnO. Field-enhanced scanning electron microscopy (FESEM) was performed to observe the stack morphology of the sensors. Figure 3a shows a cross-sectional FESEM image of the CNTs-CFH gas sensor. CNTs appear to be well deposited on the top of the 50 nm thick ZnO layer. X-ray photoelectron spectroscopy (XPS) analysis was performed to study the elemental composition of ZnO and CNTs. The chemical states and compositions were analyzed by using XPS in which C1s (284.8 eV) was utilized to calibrate the binding energies of ZnO and CNTs. The high-resolution spectrum of XPS for Zn 2p, as shown in Figure 3b, indicates that the two prominent peaks at 1021.53 eV and 1044.63 eV correspond to Zn 2p^3/2^ and Zn 2p^1/2^, respectively. The difference in binding energies between these two peaks is calculated to be ~23 eV, and this corresponds to the Zn^2+^ valance state [26,27]. Moreover, Figure 3c shows the high-resolution O1s spectra, which are deconvoluted into three distinct peaks. The peak that fitted at 494.45 eV refers to the lattice oxygen present in the form of O^2−^ in Zn-O bonding. The peak fitted at 531.13 eV refers to the oxygen vacancies, whereas the peaks located at high binding energy of 532.06 eV correspond to the chemisorbed oxygen due to hydroxyl groups. These results are consistent with the previously reported results [28]. The N1s peak of the CNTs and en-APTAS-decorated samples showed significant changes in peak intensity, as shown in Figure 3d. The XPS spectra of N1s peak of en-APTAS-decorated CNTs appeared to be intense as compared to those for bare CNTs. These results demonstrate the successful decoration of the nitrogen-based space group on the surface of the CNTs [20]. Figure 3e,f show the high-resolution XPS spectra of C1s of the CNTs and en-APTAS-decorated CNTs. In the case of bare CNTs, the C1s peaks are deconvoluted into two distinct peaks, corresponding to sp^2^ C-C graphitic carbon at 287.75 eV and the hydroxyl group at 285.83 eV. Similarly, in the case of en-APTAS-decorated CNTs, the dominant peak of graphitic carbon appeared at 287.72 and the hydroxyl group appeared at 285.67. Thus, C1s peaks of the bare CNTs and en-APTAS-decorated CNTs do not show any distinct differences in the binding energy. However, the peak intensity of C1s of en-APTAS-decorated CNTs-CFH gas sensor is observed to be decreased as compared to that of the CNTs-CFH gas sensor, which is consistent with the previously reported results [29]. To further confirm the successful coating of en-APTAS onto the surface of CNTs, the atomic percentages of C1s, O1s, and N1s were analyzed for both bare CNTs and en-APTAS-decorated CNTs. For the bare CNTs, the atomic percentages of C1s and O1s were determined to be 96.55% and 3.45%, respectively, with no detectable N1s peak. In contrast, for en-APTAS-decorated CNTs, the atomic percentages of C1s, O1s, and N1s were measured at 90.56%, 3.40%, and 6.04%, respectively, indicating successful functionalization and showing consistency with previously reported results [20].

### 3.3. Electrical Measurements

**Figure 4:**
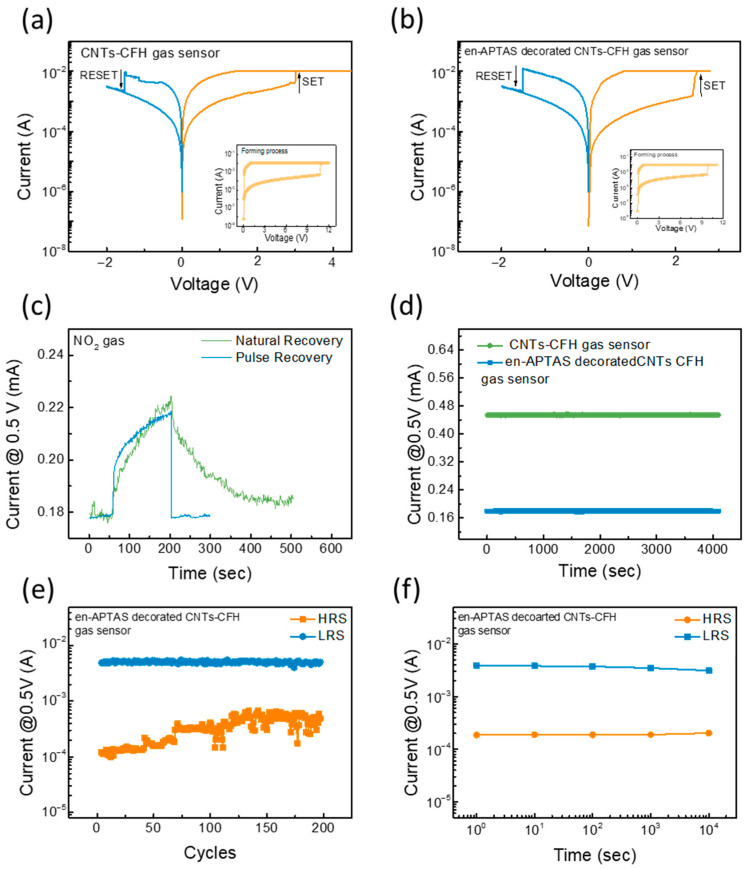
I-V curve of the gasistor with (**a**) CNTs-CFH gas sensor and (**b**) en-APTAS-decorated CNTs-CFH gas sensor. (**c**) Schematics of pulse recovery; (**d**) sensor stabilities of CNTs-CFH gas sensor and en-APTAS-decorated CNTs-CFH-based gas sensor in ambient environment under constant V_sensing_; (**e**) endurance of the en-APTAS-decorated CNTs-CFH gas sensor; (**f**) retention of 10^4^ s of the en-APTAS-decorated CNTs-CFH gas sensor.

Prior to performing the gas sensing measurements, electrical measurements were performed to determine the formation and rupture of the CF and evaluate its properties as a heat source. Figure 4a,b show the typical RS characteristics of the proposed CNTs-CFH and en-APTAS-decorated CNTs-CFH gas sensors. First, a high voltage was applied to observe the initial forming process in which migration of oxygen vacancies formed the CF under the influence of the applied voltage. The forming process was observed at 11.5 and 10.2 V for CNTs-CFH and en-APTAS-decorated CNTs-CFH, respectively. The formation of CF changed the resistance state of the device from a high-resistance state (HRS) to a low-resistance state (LRS). After the initial forming process, a negative voltage was applied to rupture the CF to obtain the HRS at reset voltages of −1.51 V and −1.53 V for CNTs-CFH and en-APTAS-decorated CNTs-CFH gas sensors, respectively. To again observe the LRS, positive voltages of 2.99 V and 2.40 V were applied to CNTs-CFH and en-APTAS-decorated CNTs-CFH gas sensors, respectively. The RS characteristics of the proposed gas sensors provide a novel approach to addressing the issue of long recovery times. Figure 4c presents a comparison between real-time recovery and pulse recovery. When CNTs interact with NO₂ gas, the current level increases due to gas reaction. Once the gas is turned off, NO₂ begins to desorb, and the current level decreases as a result of this desorption. However, this recovery process is slow, taking more than 200 s to completely recover. To achieve faster recovery, a negative pulse of −2.5 V was applied to the CFH, which accelerated the desorption of the gas and allowed the sensor to rapidly return to its initial current level. The sensor stability of both of the gas sensors was measured at V_sensing_ of 0.5 V in the ambient environment, as shown in Figure 4d. Both gas sensors showed a stable current, which remained level till 4000 s, confirming the stable initial state of the gas sensors. To ensure the reliability of the en-APTAS-decorated CNTs-CFH gas sensor, an endurance test comprising 200 DC cycles was performed at a V_read_ of 0.5 V, as shown in Figure 4e. The resistance state stability of the en-APTAS-decorated CFH-CNT gas sensor over a longer period of time was evaluated by performing the retention measurement for 10,000 s under both HRS and LRS, as shown in Figure 4f, indicating the superior stability of the sensors.

### 3.4. Gas Sensing Measurements

**Figure 5:**
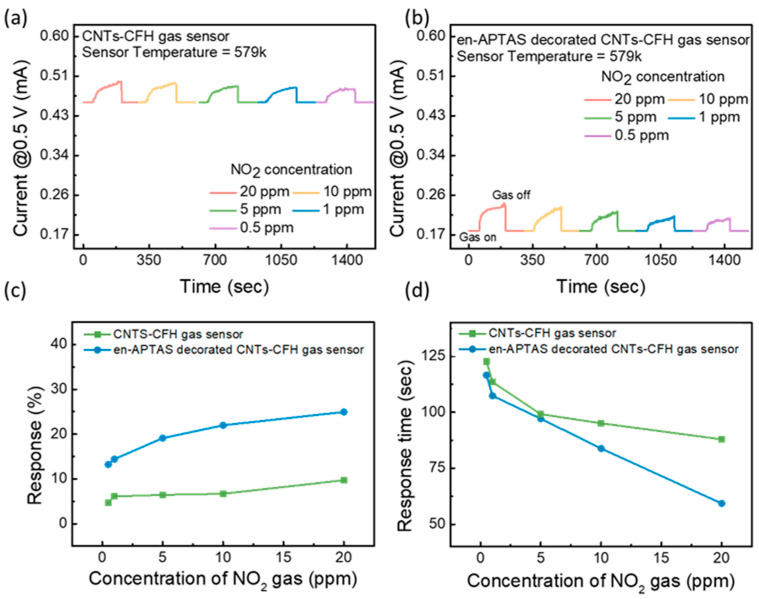
Gas sensing characteristics of (**a**) CNTs-CFH gas sensor and (**b**) en-APTAS-decorated CNTs-CFH gas sensor. (**c**) Evaluated gas sensing response % of CNTs-CFH and en-APTAS-decorated CNTs-CFH gas sensors. (**d**) Evaluated gas sensing response time of CNTs-CFH and en-APTAS-decorated CNTs-CFH gas sensors.

Measuring the gas sensing of the proposed gas sensors was performed by exposing the devices to the target gas while monitoring the change in the current value at a V_sensing_ of 0.5 V. The gas sensing measurements were performed in the HRS of both gas sensors. The HRS of the gas sensor corresponds to the high resistance state, which results in a high temperature inside the CFH. This high temperature will be used as a nano-heater to observe the enhanced gas sensor characteristics at room temperature. The gas sensing measurements were performed to evaluate the sensitivity of the proposed gas sensors towards different concentrations (20–0.5 ppm) of the NO_2_ gas. Figure 5a,b show the transient curve of the CNTs-CFH and en-APTAS-decorated CNTs-CFH gas sensors. The gas sensing results show the instant change in the current level as the target gas is injected into the chamber. The change in the current level resulted from the reaction between the surface of the CNTs and the NO_2_ gas. The change in the current levels of both the gas sensors showed a direct relation with the concentration of NO_2_ gas. Equation (1) is used to calculate the response percentage of the gas sensor. The calculated response percentage of the CNTs-CFH gas sensor and the en-APTAS-decorated gas sensor is shown in Figure 5c. The response of the en-APTAS-decorated CNTs-CFH gas sensor is observed to be higher than that of the CNTs-CFH gas sensor at all concentrations of NO_2_ gas. Figure 5d shows the response time plot of CNTs-CFH and en-APTAS-decorated CNTs-CFH gas sensors for all the concentrations of NO_2_ gas. The response time increases with the decrease in the concentration of NO_2_ gas, which is due to the low diffusion rate and slow reaction at low concentrations of NO_2_ gas with the CNTs. The response time of the en-APTAS-decorated CNTs-CFH gas sensor is observed to be faster than that of the pure CNTs-CFH gas sensor, which again confirms that the modified CNTs surface offers more reaction sites and high reactivity towards NO_2_ gas.

**Figure 6:**
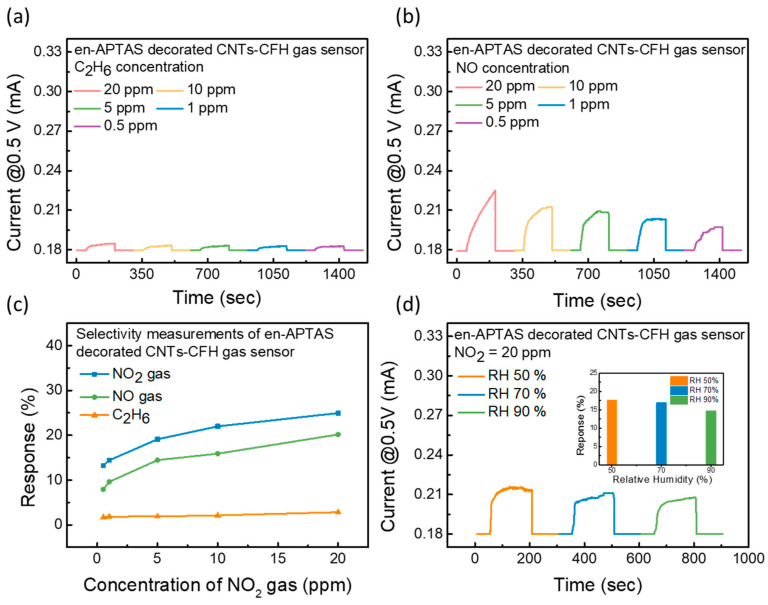
(**a**) Transient response of gas sensing measurements of C_2_H_6_ gas and (**b**) NO gas to evaluate the selectivity of the gas sensors; (**c**) evaluated response percentages comparison with response percentage of NO_2_ gas; (**d**) transient response curves of en-APTAS-decorated CNTs-CFH gas sensor at relative humidity levels of 50%, 70%, and 90%. Inset shows the evaluation of response degradation due to relative humidity effect.

To further check the superiority of the proposed en-APTAS-decorated CNTs-CFH gas sensor, selectivity measurements were performed towards NO and C_2_H_6_ gases, as shown in Figure 6a,b. In addition to NO, C_2_H_6_ gas was chosen for the selectivity measurements because it is a representative gas of the hydrocarbon group. C_2_H_6_ is extensively used in industries for the development of plastic materials [30] and it is also present in exhaled human breath [31]. Thus, detection of C_2_H_6_ gas extends the detection capabilities of the proposed en-APTAS-decorated CNTs-CFH gas sensor. Selectivity measurements were performed with 20–0.5 ppm of the target gases. Furthermore, a comparison of response percentages for NO_2_, NO, and C_2_H_6_ is shown in Figure 6c. The en-APTAS-decorated CNTs-CFH gas sensor showed the lowest response towards C_2_H_6_ gas due to its low adsorption energy. CNTs-based gas sensors have frequently been utilized for the detection of NO gas. However, the en-APTAS-decorated CNTs-CFH gas sensor showed a significantly high response towards NO_2_ compared to NO gas. The increase in the response towards NO_2_ gas is related to the larger kinetic diameter of NO_2_ gas, which is reported to be 3.83 Å [32,33]. Due to the larger kinetic diameter of NO₂ gas, it tends to have stronger interactions with both CNTs and en-APTAS. These results show the superiority of the selectivity measurements of the sensor towards NO_2_ gas. The effect of relative humidity (RH) was evaluated by increasing the RH level. Figure 6d shows the transient gas sensing curves of the en-APTAS-decorated CNTs-CFH gas sensor at 20 ppm of NO_2_ gas by maintaining an RH range of 50–90%. The response percentages at different RH levels are plotted in the inset of Figure 6d, indicating a 39% degradation in the response percentage at 90% RH as compared to the response percentage at 30% RH. The en-APTAS-decorated CNTs-CFH gas sensor showed fairly stable response characteristics due to the heating provided by the CFH at a V_sensing_ of 0.5 V at an elevated RH level. These results suggest that heating the CNTs using CFH provided resistance in the adsorption of water molecules and validated the stability of the en-APTAS-decorated gas sensor towards a high RH level.

Table 2 presents a comparative analysis of this study’s findings with previous research on gas sensors utilizing CNTs materials. Despite these advantages, ongoing efforts are directed toward improving their ability to detect a wider range of gases, often through the integration of supplementary devices like heaters. In this study, a CNT gas sensor was combined with a CFH. Unlike conventional heaters, CFH enhance the sensing capabilities of the proposed CNT gas sensors. Consequently, the proposed gas sensor demonstrates a competitive performance compared to those presented in prior studies, achieving a response of 24.94 at 20 ppm for the en-APTAS-decorated CNTs-CFH gas sensor and 9.72 for the bare CNTs-CFH gas sensor. Additionally, the incorporation of a CFH significantly improved the recovery time, reducing it to just 1 millisecond. This addresses the challenge of slow recovery and enables CNTs to effectively return to their initial state.

### 3.5. Gas Sensing Mechanism

**Figure 7:**
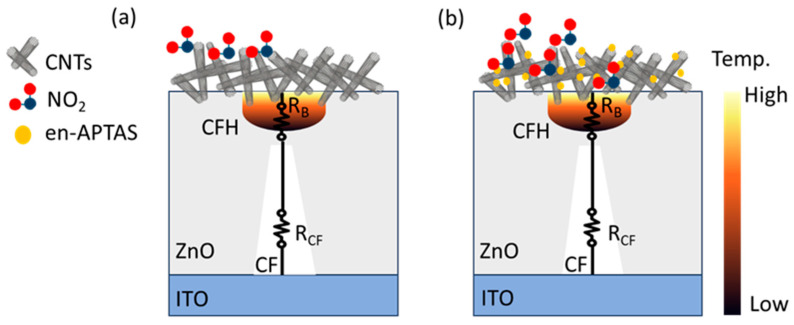
Schematics of interaction of NO_2_ gas with (**a**) CNTs and (**b**) en-APTAS-decorated CNTs.

The gas sensing mechanism of the CNTs-CFH gas sensor operates through charge transfer when NO₂ molecules are adsorbed and desorbed onto the sensor’s surface. Prior to performing the gas sensing measurements, the HRS of the CFH was achieved by rupturing the CF. When a negative voltage is applied, the CF ruptures due to the Joule heating effect. At the HRS, two types of resistances appear inside the CFH, i.e., (i) resistance of the ruptured CF (R_CF_) and (ii) resistance of the bulk (R_B_), which appeared at the gap of the ruptured CF. On the application of a sensing voltage of 0.5 V, maximum power dissipation occurs at the bulk resistance (R_B_), resulting in an increase in the heat. This generation of heat is used as a nano-heater to observe the gas sensing characteristics at RT. Figure 7a,b show the interaction of the NO_2_ gas with the CNTs and the en-APTAS-decorated CNTs. Due to their natural abundance of holes, CNTs function as p-type semiconductors, while NO_2_, being an excellent oxidizing agent, tends to capture electrons [39]. In ambient conditions, oxygen interacts with the CNT surface, capturing an electron and creating an $O_{2}^{-}$ depletion layer on the CNT surface. The removal of electrons from the CNT surface increases the number of holes, which facilitates electric current conduction. When the CNTs are exposed to NO₂ gas, the chemisorbed $O_{2}^{-}$ ions react with NO₂ gas molecules, leading to the formation of numerous ${NO}_{2}^{-}$ species. These reactions can typically be described as follows [40]:

(5) $$O_{2}+e^{-}\to O_{2}^{-}$$

(6) $$O_{2}+e^{-}\to {2O}^{-}$$

(7) $${NO}_{2}+e^{-}\to {NO}_{2}^{-}$$

(8) $${NO}_{2}+O_{2}^{-}+2e^{-}\to {NO}_{2}^{-}+2O^{-}$$

The increase in the gas sensing response of the en-APTAS-decorated CNTs-CFH gas sensor is due to increased adsorption energy of the functionalized surface of CNTs. Furthermore, the amine group attached to the surface of CNTs results in the occurrence of electron-abundant adsorption sides, which resulted in the enhanced gas sensing response. Thus, in conclusion, these findings suggest that functionalization of the CNTs surface is an adoptable method to improve the gas sensing response towards NO_2_ gas.

## 4. Conclusions

CNT gas sensors are considered to be potential candidates for the detection of NO_2_ gas. However, CNTs still face various issues related to sensitivity and slow recovery. The proposed gas sensor is decorated with an en-APTAS membrane and embedded with a CFH as a heater to resolve these issues. An enhanced gas sensing response of 24.9% was achieved for the en-APTAS-decorated CNTs-CFH gas sensor, which is 60% higher than the undecorated CNTs-CFH gas sensor. Furthermore, fast recovery is achieved by applying a pulse of −2.5 V, with a pulse width of 1 ms, to the CFH. The application of high voltage resulted in the quick desorption of NO_2_ gas due to the occurrence of an elevated temperature inside the CFH. Moreover, CFH also improved the effect of RH levels of the proposed gas sensor, as the en-APTAS-decorated CNTs-CFH gas sensor showed a degradation of 39% in the response value at the 90% RH level. Thus, the proposed methods offer a possible solution to resolve the drawbacks of CNT gas sensors.

## Figures and Tables

**Table 1:** Material parameters used in the model [24,25].

Material	V_sensing_	K [WK^−1^m^−1^]	C_p_ [JKg^−1^K^−1^]	σ [Sm^−1^]	ԑ_r_	*ρ* [Kgm^−3^]
ZnO (with en-APTAS)	0.5 V	49	40.3	3.19 × 10^−4^	2.4	5.606 × 10^3^
ZnO (without en-APTAS)	0.5 V	49	40.3	3.39 × 10^−4^	2.4	5.606 × 10^3^
ZnO_1−x_	-	70	100	2 × 10^−4^	3	6.500 × 10^3^

**Table 2:** Comparison of CNT-based gas sensors for NO_2_.

Sensing Gas	Temperature	Sensing Material	Concentration of Gas (ppm)	Response %	Recovery Time (s)	Ref
NO_2_	MHH in HRS	CNT	10	1.46	0.001	[13]
NO_2_	MHN in HRS	CNT	10	1.77	0.001	[13]
NO_2_	Room temperature	B-doped CNT/SnO_2_	2	2	9000	[34]
NO_2_	Room temperature	rGO-CNT SnO_2_	5	2.53	77	[35]
NO_2_	Room temperature	Mesh-shaped MWNT	50	2.28	N/A	[36]
NO_2_	Room temperature	PPy/N MWCNT	5	1.25	668	[37]
NO_2_	Room temperature	SWCNT/SnO_2_	600	71	90	[38]
NO_2_	CFH in HRS	CNT	50	54	0.001	[21]
NO_2_	Room Temperature	HfO_2_	25	22	N/A	[23]
NO_2_	Room Temperature	CNTs-TE with en-APTAS	10	5.32	0.000006	[19]
NO_2_	CFH in HRS	CNTs-CFH gas sensor	20	24.94	0.001	This work
NO_2_	CFH in HRS	En-APTAS coated CNTs-CFH gas sensor	20	9.72	0.001	This work

## Data Availability

The data are contained within the article.
